# Beyond the bilayer: multilayered hygroscopic actuation in pine cone scales

**DOI:** 10.3762/bjnano.16.119

**Published:** 2025-09-29

**Authors:** Kim Ulrich, Max David Mylo, Tom Masselter, Fabian Scheckenbach, Sophia Fischerbauer, Martin Nopens, Silja Flenner, Imke Greving, Linnea Hesse, Thomas Speck

**Affiliations:** 1 Cluster of Excellence livMatS @ FIT—Freiburg Center for Interactive Materials and Bioinspired Technologies, University of Freiburg, Freiburg im Breisgau, Germany; 2 Plant Biomechanics Group @ Botanic Garden, University of Freiburg, Freiburg im Breisgau, Germany; 3 Department of Microsystems Engineering—IMTEK, University of Freiburg, Freiburg im Breisgau, Germany; 4 Biomimetics Group, Institute for Wood Sciences, University of Hamburg, Hamburg, Germany; 5 Thünen Institute of Wood Research, Hamburg, Germany; 6 Institute of Materials Physics, Helmholtz-Zentrum Hereon, Geesthacht, Germany

**Keywords:** digital volume correlation (DVC), finite element analysis, hygroscopic bending, plant biomechanics, sorption measurements

## Abstract

The anisotropic hygroscopic behavior of pine cone scales and its effect on bending motion, with implications for bioinspired actuation, is investigated. Using gravimetric water uptake measurements, synchrotron radiation-based nano-holotomography, and digital volume correlation analysis, inter- and intra-tissue variations of hygroscopic swelling/shrinkage were observed. In addition, the moisture content of pine cone scale tissues was measured as a function of relative humidity. There were distinct differences between tissues and a pronounced hysteresis between sorption and desorption. Finite element analysis was performed on geometries ranging from simplified bilayer models to complex remodeled scales. Simulation results showed an underestimation of the bending of bilayer geometries due to an overestimated contribution of sclerenchyma fiber stiffness. Geometries with discrete fibers embedded in a brown tissue matrix more accurately reproduced the bending angles observed in experiments. This highlights the importance of the chosen material properties and tissue arrangements for predicting pine cone scale bending in silico. By contributing to a deeper understanding of pine cone scale biomechanics, these results also support the development of bioinspired technical applications. Future studies should refine tissue mechanical properties and integrate high-resolution computed tomography-based geometries to further elucidate the mechanisms underlying hygroscopic actuation. This integrative approach will bridge experimental findings with computational modeling and advance plant biomechanics and biomimetic transfer.

## Introduction

In recent years, the hygroscopic motion of plant structures has attracted increasing attention from researchers and engineers, both for fundamental biological research and bioinspired technical applications [1–4]. Of particular interest are, for example, *Banksia* seed pods [5–6], *Hakea* fruits [7–8], and scales of pine cones [9–12], which passively respond to changes in ambient relative humidity by shape morphing to facilitate seed dispersal. In the case of pine cones, winged seeds are blocked and protected by the scales under wet conditions and released under dry conditions for wind dispersal. The inspiration provided by such biological motion principles has driven innovation in a variety of fields, including wood elements for weather-responsive and self-forming building parts [13], ceramic bilayer actuators [14], and 4D-printed weather-responsive façade shading elements [15]. The underlying principle in these biological examples is often compared to a thermally actuated “bimetal” mechanism [16–17]. The differential expansion or shrinkage of adjacent tissues drives the bending motion. However, current research on pine cones is increasingly revealing more complex, multilayered systems than simple bilayer analogies suggest. For instance, isolated pine cone scale tissues can bend independently, highlighting the complexity and resilience of the system despite delamination or cracking [18–19]. Additionally, research concerning the initial cone opening [20] and scale opening orchestration [21] has been conducted, highlighting the functional robustness and resilience [22] of pine cones. A better understanding of the pine cone scale and how its tissue properties and arrangement impact the bending motion, can therefore benefit the development and improvement of novel bioinspired technical applications.

At present, some characteristics of pine cones have been described that help to explain how their individual tissues can deform under desiccation/hydration and thus lead to the bending motion of the scale. One crucial influencing factor is the difference within or between tissues, as Eger et al. [23] highlighted. The authors measured the relative change of moisture content as a function of relative humidity by gravimetric water uptake measurements of the sclereid cells, the brown tissue, and the sclerenchyma fibers of a *Pinus wallichiana* pine cone scale. However, as their water uptake measurements consisted of only one measurement per tissue, being limited to the range of 30–80% RH, and their equilibrium criterion being one hour of exposure instead of a mass change threshold, a more nuanced study of the differences in water uptake between tissues is required to gain a better understanding of motion actuation. Quan et al. [24] also measured the gravimetric water uptake, revealing a hysteresis between uptake and evaporation, but without distinguishing between the scale tissues. Furthermore, they described a porosity gradient in the sclereid cell layer, without distinguishing between the sclereid cell layer and the brown tissue layer. The more porous adaxial sclereid cells they described are consistent with the earlier description of the brown tissue between the sclereid layer and the sclerenchyma fibers [12]. Other approaches have also observed a gradient in the cross-sectional shape of the prosenchymatous cells of sclerenchyma fibers of *Pinus elliottii* cones and higher tensile strength of the abaxial compared to the adaxial side of the sclerenchyma fibers [18].

Although recent advances in the study of pine cone scales have revealed the involvement of more than two tissue layers (sclereid cells, brown tissue, and sclerenchyma fibers) that may themselves contain a gradient [18–19,23–24], the relationship between these gradients and the resulting axis-dependent hygroscopic expansion of the tissues is still not described. To date, the hygroscopic expansion of pine cone scale tissues was often described under the assumption of isotropic expansion. Dawson et al. [11] measured the hygroscopic expansion coefficient of *Pinus radiata* cone scale tissues, but since then, an axis-dependent description of the hygroscopic expansion has remained omitted. This axial dependence of hygroscopic expansion has been an important research topic in wood research for years [25–27], but has so far been widely neglected in research on hygroscopic plant movements.

Recent advancements in the application of digital volume correlation (DVC) [28] in combination with synchrotron radiation-based nano-holotomography enable the study of axially dependent hygroscopic expansion and shrinkage of tissues [29]. This approach overcomes previous limitations related to the use of only affine registration or the previously neglected longitudinal (along the cell length axis) expansion of the measured cells [26–27]. By scanning tissue samples in a moist and a dry state using nano-holotomography, a DVC-based analysis of the two states becomes possible, which results in a displacement field representing the deformation from one state to the other. Using this displacement field allows for the calculation of a strain tensor field, which describes the axis-dependent hygroscopic expansion [29].

Incorporating the anisotropic swelling and shrinkage behavior into in silico models of the pine cone scale system further facilitates a more precise description of the bending motion. When simulating and analyzing the hygroscopic bending of pine cone scales using finite element analysis (FEA), as has already been done for a bilayer system [23,30], some geometrical simplifications and assumptions have to be made. For example, the cross-sectional shape of the tissues is simplified to rectangular shapes and modeled solely as a bilayer, which hardly mimics the scales. With these simplifications, a bending motion can be simulated, but the conclusions that can be drawn, for example, regarding the impact of the tissue involved and their arrangement, are very limited.

The aim of this work is to characterize the anisotropic hygroscopic behavior of pine cone scale tissues and its effect on the bending motion. To this end, we first characterize their sorption behavior using gravimetric water uptake measurements. Furthermore, we apply synchrotron radiation-based nano-holotomography paired with DVC analysis to measure the axial dependent dimensional changes of cell size level tissue samples while shrinking. Finally, we compare measurements of the opening angle of pine cone scales with FEA models of different abstraction levels of a pine cone scale cross-sectional geometry incorporating the DVC-estimated hygroscopic expansion coefficients. This will answer the questions of (1) whether an intra-tissue gradient of the hygroscopic strain can be observed, (2) whether this measured hygroscopic strain can be used in a FEA to reproduce a comparable bending motion, and (3) whether this will allow us to properly describe and explore the effect of the cross-sectional shape and arrangement of the tissues on the bending motion.

## Experimental

### Sample preparation

All four pine cones used in our experiments originated from a *Pinus jeffreyi* tree growing in the Botanical Garden in Freiburg im Breisgau, Germany, and were collected from the ground in February 2022, after they had already opened and dispersed most of their seeds. Following collection, the cones were stored in a climate-controlled environment at room temperature. If individual cone scales were required for experiments, a pine cone was submerged in water overnight and then the scales were removed from the central axis by hand.

#### Gravimetric water uptake

**Figure 1:**
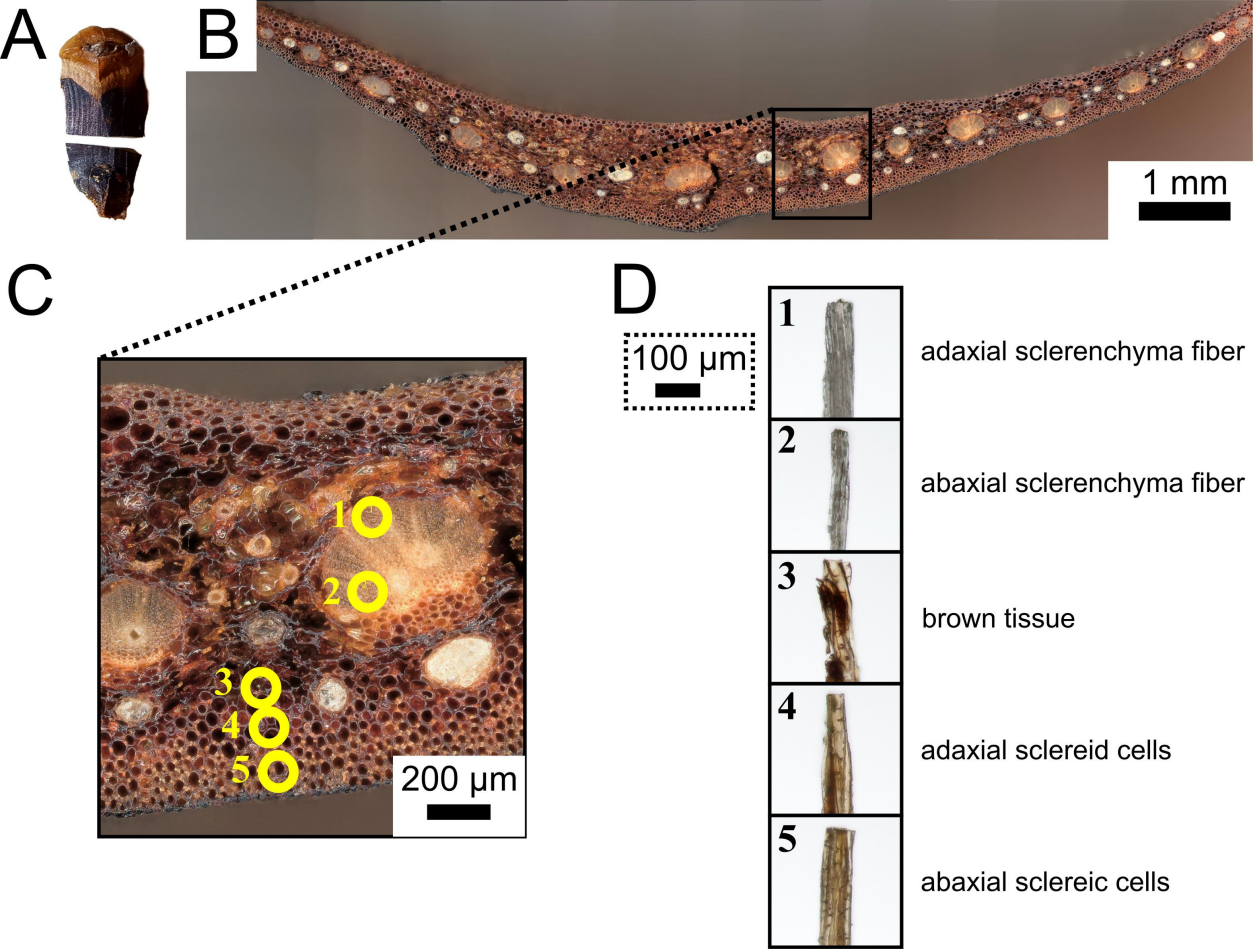
Tissue sample extraction from a *Pinus jeffreyi* cone scale. (A) The position of the cross section along the longitudinal axis of the scale, where the tissue samples were prepared from. (B) The respective scale cross section at the sampling location. (C) Close-up of the exact sampling locations within the tissues. (D) Microscopic images of the upper end of the respective tissue sample pillars.

The following three tissues were isolated from ten scales of the first cone: (1) the sclereid cell layer with the abaxial epidermis, (2) the brown tissue, and (3) the sclerenchyma fibers (Figure 1). Each scale was first saturated with water to facilitate subsequent separation with a razor blade. Saturating the scales with water prior to the sorption experiments prevents measuring a biased scanning isotherm due to the unknown sorption history of the sample [31]. We then divided the total amount of separated tissue material equally into five sample dishes each. The gravimetric water uptake of the extracted samples was then measured using a sorption test system (SPSx-1µ-High-Load, ProUmid, Germany) and a temperature and humidity sensor (HC2A-S Ambient Air Probe, ROTRONIC, Germany). At a controlled temperature of 20.0 °C, the samples were exposed to different steps of relative humidity, starting at 90%, dropping to 0% in increments of 15%, and returning to 90%, respectively. During this time, the samples were weighed every 20 min until all samples reached equilibrium. Equilibrium was defined as a change of mass of less than 0.01% over a period of 40 min. The 0% RH climate step was maintained for at least 96 h to ensure an equilibrium when measuring the dry mass of the samples. During the measurements, an error occurred in one sample of sclerenchyma fiber tissue, resulting in a pronounced mass shift from one weighing step to the next, making it unsuitable for further analysis. The relative mass change of the samples with respect to the measured dry mass was calculated and used for further analysis.

#### Axially dependent hygroscopic tissue shrinkage

The general imaging and analysis protocol followed the procedure presented in Ulrich and colleagues [29]. A pine cone scale was isolated from the second of the collected cones and transported to Hamburg, Germany, where the scale was soaked in water overnight to improve its cutability. Tissue samples were prepared of the ab- and adaxial parts of the sclereid cell layer and the sclerenchyma fibers, and the brown tissue (Figure 1). Each sample was cut into small pillars (length: ~800 µm, width: ~50 µm) by hand using a microscope (Olympus BX51, Evident Europe GmbH, Hamburg, Germany) and a razor blade. One end of the pillars was then glued to special sample holders using a UV-cured liquid plastic welding system (Bondic BC4000, VIKO UG, Kranzberg, Germany). Each tissue sample was then imaged at a special synchrotron radiation-based nano-holotomography setup at the imaging beamline P05 operated by Helmholtz-Zentrum Hereon at PETRA III (DESY Deutsches Elektronen Synchrotron, Hamburg, Germany). Phase contrast-based near-field holotomography was used to image our samples, utilizing a 300 µm gold Fresnel zone plate to focus the monochromatic beam with an energy of 11 keV [32]. For the in situ nano-holotomography, a climate chamber [33] was used to image the specimen in a moist (90% RH) and a dry (<3% RH) state. A binning factor of two was applied to the images. The phase retrieval was performed using the Holowizard framework [34–35]. The sample volumes were reconstructed using the GridRec algorithm [36] with a Shepp–Logan filter, implemented in TomoPy [37] with the P05 reconstruction pipeline. The resulting voxel size was 127 nm.

Prior to the DVC analysis, some preprocessing was performed using FIJI (ver. 1.54f) [38]. Since the sample expanded in vertical direction, the imaged sample area is not exactly the same in both scans. Therefore, the images were cropped to the same sample region for comparability. Additionally, the bit type of the images was reduced to 8-bit for computational efficiency, which reduced the computational time with negligible loss of information.

DVC was performed using the image registration software Elastix with its implemented B-spline transformation (ver. 5.1.0) [39]. The images were aligned in a two-step process, first with an affine transformation and then with a B-spline transformation. For both steps, a pyramidal approach with five resolution levels using a Gaussian filter was applied. The number of iterations per resolution level was set to 1000 for the affine transformation and 2000 for the B-spline transformation. Advanced normalized correlation was chosen as the correlation metric. The detailed parameter files, “Affine_parameters.txt” and “BSpline_parameters.txt” can be found in Supporting Information File 1. Based on the resulting displacement field, the Green–Lagrangian strain was calculated using the Insight Toolkit strain filter extension (ITK: ver. 5.3.0, itk-strain: ver. 0.4.0) [40] in Python (ver. 3.11.9) [41]. Finally, for each sample, the median of the axial strain components across all sample bulk voxel entries was calculated.

#### Hygroscopic bending motion

**Figure 2:**
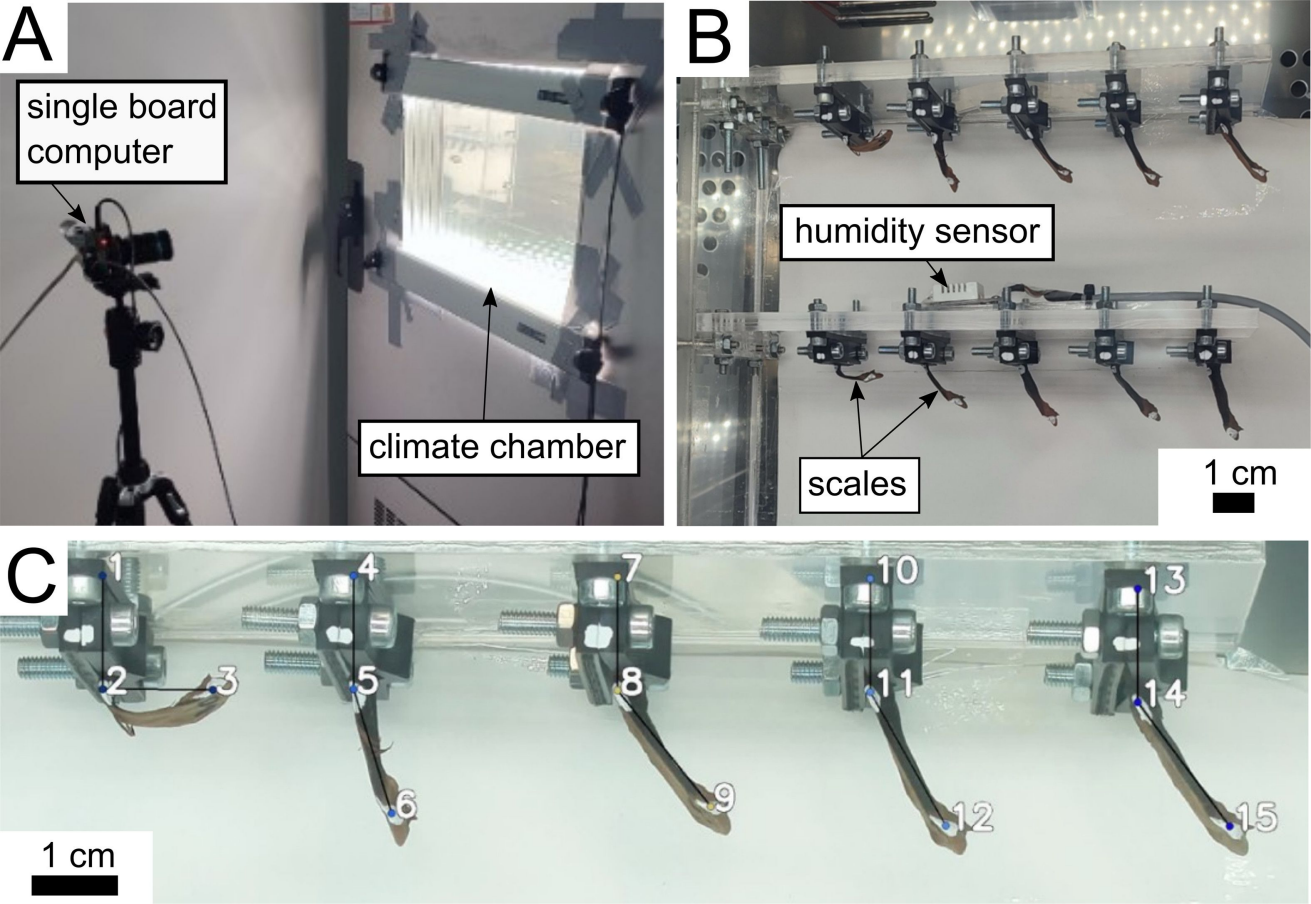
Characterization of the scale bending angle as a function of relative humidity and temperature. (A) Single board computer with camera module in front of the climate chamber. (B) Ten *Pinus jeffreyi* cone scales fixed in a sample holder inside the climate chamber. A row of scales of one cone is sorted from more basal (left) to more apical (right) based on the original position along the central cone axis. (C) Single frame of the first scale row during the automated point tracking for calculating the bending angle.

The scales of the last two pine cones were isolated, and two of the scale rows organized in steep spirals along the central axis were randomly selected for both cones. From each spiral, five scales were prepared for measurement, starting with the first basal scale whose adaxial epidermis was not blocked by another scale. For each experiment, one set of ten scales from one cone was then mounted in two rows of five in a sample holder inside a climate chamber (CTC-265, Memmert GmbH + Co.KG, Schwabach, Germany), with the rows representing one of the cone spirals. The most basal scale was mounted on the left and the most apical scale on the right (Figure 2). For further analysis, the position along the scale axis was assigned to an ordinal scale, with 1 being most basal and 5 being most apical. The scales were mounted by clamping the leftover part of the central cone axis at the basal end of the scale. The basal and apical parts of these scales facing the window were marked with a larger white dot with a black dot in the center to improve visibility and traceability.

To test how long it takes for the scale bending motion to equilibrate, we set the climate chamber to run for 48 h, with the first 24 h at 31.7 ± 0.2% RH and the second 24 h at 73.9 ± 0.9% RH. The time to equilibrium was then calculated for each climate step. Equilibrium was defined as an angular change of less than 0.01° over 60 min. In a second run, the climate chamber was programmed to start at 30% RH and remain there for 24 h, then increased to 75% RH in four ~12% increments. Each incremental climate step was held for 12 h. After remaining at the highest humidity level for 24 h, the RH was again lowered back to 30% in four 12 h long decrements. Due to the limitations of the climate chamber and the required large volume of the interior, the equilibration steps achieved could vary between the incremental increases and decreases in humidity. The temperature and humidity in close proximity to our samples inside the chamber, as well as a side view of the samples, were recorded externally every 10 min using a single-board computer (Raspberry Pi 4 Model B, Raspberry Pi Foundation, Cambridge, UK) connected to a temperature and humidity sensor (DHT22, Sertronics GmbH, Berlin, Germany) and a camera module (Raspberry Pi HQ Camera V1.0, Raspberry Pi Foundation). The captured images were then analyzed using the adapted tracking script by Cheng et al. [15] to measure the opening angle of the scales. This was done by calculating the angle between a vertical line passing through the base of a scale and the connecting line between the base and apical points. The average of the last five angles measured for each climate step was then used for further analysis. The angle attained at equilibrium during the initial drying step was used as the baseline for normalizing all angle measurements.

#### Scale bending simulation

**Figure 3:**
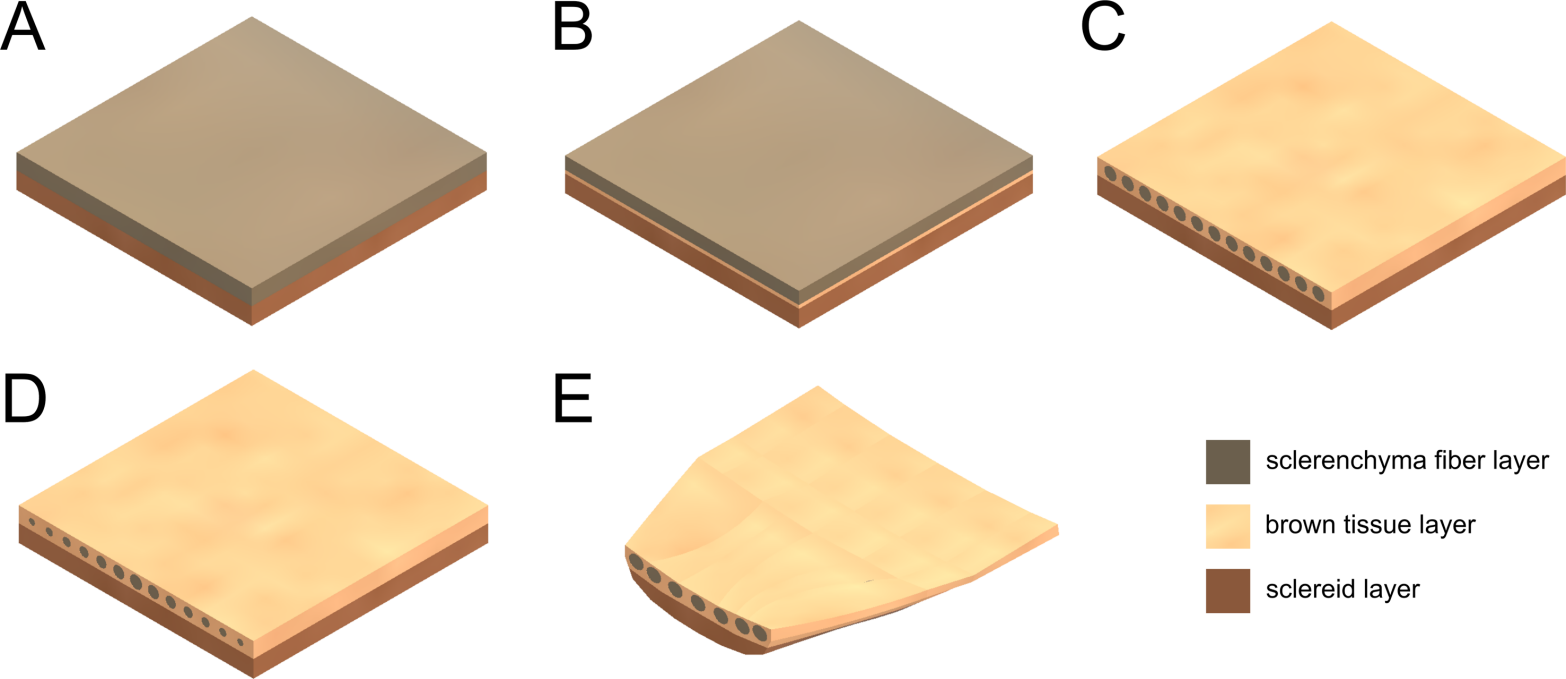
Geometries used for finite element analysis. (A) Bilayer. (B) Trilayer. (C) Trilayer with uniform fibers. (D) Trilayer with varying fibers. (E) Remodeled scale (“scale-like”).

Based on a CT scan of a *P. jeffreyi* cone scale bending zone (basal third), five CAD geometries were created (Figure 3). The geometries were kept within the same dimensions (length: 11.5 mm, width: 11.5 mm, and thickness: 1.6 mm), to compare the resulting bending motion with the bending of the natural scales. The first geometry was a bilayer consisting of the sclereid layer and the sclerenchyma fiber layer, both being modeled with rectangular cross sections. The second geometry was a trilayer with the addition of a (thinner) intermediate brown tissue layer. The third geometry was a trilayer with the sclerenchyma fibers being modeled as uniform tubes within the brown tissue layer. The fourth geometry was similar to the third, but with the diameter of the sclerenchyma fiber tubes gradually decreasing from 0.6 mm medial to 0.3 mm lateral. The last geometry resembled the natural anatomy of the pine cone scale the most as the geometry was modeled on the basis of five sketches of corresponding CT cross sections from five distinguished locations with defined distance along the longitudinal scale axis. Starting with a cross section from the basal part of the bending zone, the following cross sections were taken from positions 2.5, 5.5, 7.5, and 11.5 mm more apical. These five cross-sectional positions were then used as control points with the same longitudinal spacing between them to extrude the volume model. Since the sclerenchyma fibers branch along the longitudinal axis of a scale, they were simplified by allowing branching only at the cross sections used as control points (Figure S1, Supporting Information File 2).

The response of the involved tissues of the five scale-derived geometries during expansion and the influence of the involved tissues were characterized by simulative FEA in Ansys (ver. 2024 R2, Ansys, Inc., Canonsburg, PA, USA). Expansion was modeled using thermal stress analysis (a combination of the steady-state thermal and static structural solvers). The thermal expansion coefficients of the three tissues involved (sclereid layer, sclerenchyma fibers, and brown tissue) were defined to be orthotropic and derived from the hygroscopic shrinkage of the individual tissues measured in section “Axially dependent hygroscopic tissue shrinkage”. The average value of the respective ab- and adaxial values was used for the sclereid layer and the sclerenchyma fibers. For the *X*- and *Y*-directions (spanning the cross section of each model), the mean value of the two measured values was used, while the *Z*-value (from the basal to the apical end of the models) was derived directly. The same coefficients were used in all simulations. A linear elastic material model was applied for all tissues, with Young’s moduli of 30 MPa for the sclereid layer, 500 MPa for the sclerenchyma fibers, and 50 MPa for the brown tissue, and a Poisson’s ratio of 0.3 for all tissues (all values derived from Eger et al. [23])*.* These five simulations will be referred to as the standard simulations in the following. To analyze the influence of the mechanical properties, in another simulation of the bilayer, the value of the Young's modulus of the sclerenchyma fibers was adjusted to that of the brown tissue (from 500 to 50 MPa). To analyze the influence of the Young's modulus of the brown tissue also in the scale-like model, another simulation was performed in which the Young's modulus was reduced below the Young's modulus of the sclereid layer from 50 to 10 MPa.

The imported geometries were converted into a volume mesh in Ansys (quadratic tetrahedrons; Tet10 elements) with edge lengths of 0.10 to 0.15 mm, resulting in meshes of 772,934 to 1,234,185 nodes (500,074 to 808,140 elements; Figure S1, Supporting Information File 2). To avoid highly deformed elements near the fixed plane, non-linear adaptive remeshing based on the skewness values of the elements was included where necessary. At the basal end of the geometry, a fixed support was applied over the entire cross-sectional area to suppress movement at this end and mimic experimental conditions. Expansion was induced by a temperature change of 150 °C in all volumes and simulated nonlinearly, accounting for large deformations. This temperature should be noted as being used only as an analogue for the hygroscopic expansion actuation, with no relation to the physical effect that an actual temperature would have on the scales. The choice of 150 °C was determined iteratively to produce a bending of the geometries in the range of the natural scale. Initially, the chosen expansion coefficients were determined so that a temperature change of 100 °C corresponds to the results of the tissue shrinkage experiment. However, it is known from preliminary experiments that the influences of individual materials in multimaterial systems affect each other and that the actuation temperature must therefore be adapted. The results of all five geometries were compared in terms of their maximum total displacement at the apical tip and the observed double curvature. For this purpose, the maximum displacement in the *Y*-direction was quantified at three points. These were located at the two adaxial apical corners of each geometry and at the adaxial apical center (Figure S2, Supporting Information File 2). In order to assess the sensitivity of the FE analyses with respect to the expansion coefficients in the measured ranges, two comparative simulations were performed on the geometry with graded fiber size. In these simulations, the expansion coefficient of the brown tissue was set to the reciprocal of the minimum or maximum measured median strains (−5.8% and −10.5%, respectively) determined by DVC.

#### Statistical analysis

Data management, statistical analyses, and visualization were done with Python (ver. 3.12.8) using the packages pandas (ver. 2.2.3) [42], scipy (ver. 1.14.1) [43] and seaborn (ver. 0.13.2) [44]. The threshold of statistical significance was set at *p* < 0.05.

## Results

### Gravimetric water uptake

**Figure 4:**
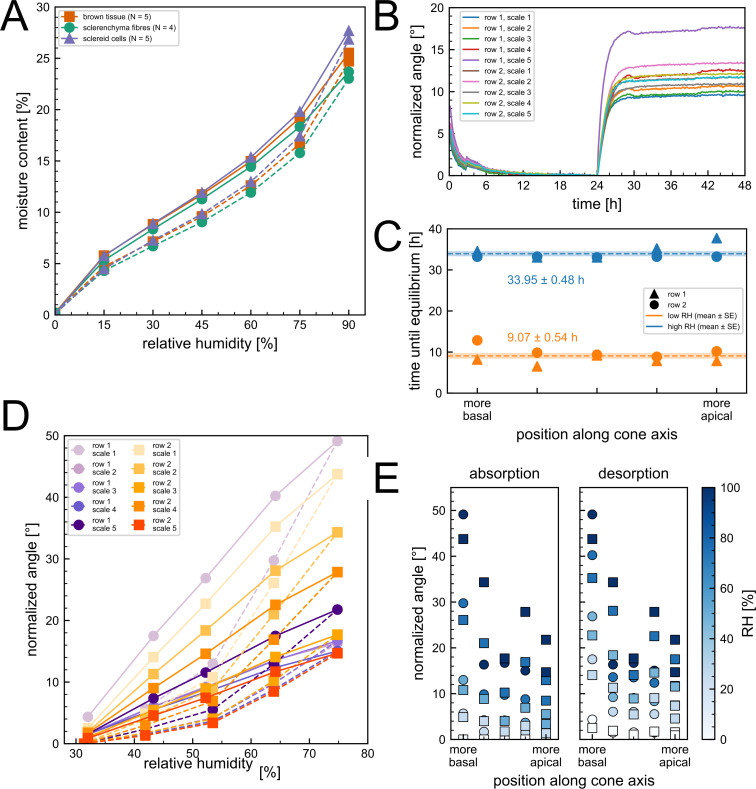
Results of the sorption measurements and the bending angle characterization. (A) The average moisture content of the scale tissues as a function of relative humidity. The desorption is shown as a solid line, the absorption as a dashed line. Violet: sclereid cells, orange: brown tissue, green: sclerenchyma fibers. (B) Ten pine cone scales where exposed to first a low, then a high relative humidity. Their bending angle normalized to the angle before changing conditions after 24 h is shown as a function of time. (C) The time it took the scales to reach equilibrium is plotted as a function of their position along the longitudinal central cone axis. Orange: low RH, blue: high RH. Circle: scales of first row, square: scale of second row. (D) Incremental measurements of the scale bending angle as a function of RH. The desorption is shown as a solid line, the absorption as a dashed line. The rows of scales are separated by color scheme. Violet: first row, orange: second row. The scales position along the central cone axis is shown by saturation. High saturation: more apical, low saturation: more basal. (E) The measured angle during absorption and desorption depicted in (D) is shown as a function of the scales position along the central cone axis. The respective RH is indicated by color. The first row of scales is depicted as circles, the second row as squares.

The gravimetric water uptake measurements allowed us to make precise analyses of the moisture content (Figure 4A). The behavior of the sclereid cells and the brown tissue revealed marked differences particularly above 50% RH. During absorption at 60% RH, the moisture content of the brown tissue is 0.37 percentage points lower than that of the sclereid cells, when looking at absolute differences. At 90% RH, the moisture content of the brown tissue is 2.13 percentage points lower. The sclerenchyma fibers have a lower moisture content than the other two tissues even at lower RHs. At 30% RH during absorption, their moisture content is 0.43 percentage points lower than the brown tissue and 0.55 percentage points lower than the sclereid cell moisture content. In the high RH range, it is particularly striking that the sclerenchyma fibers absorb the least percentage of water, the sclereid cells the most, and the brown tissue in between. At 90% RH, the sclereid cells achieve a moisture content of 27.7 ± 0.4% for desorption and 26.8 ± 0.4% for absorption. In comparison, the sclerenchyma fibers achieve a moisture content of 23.7 ± 0.6% for desorption and 23.0 ± 0.5% for absorption. In terms of the hysteresis observed, the difference in moisture content between desorption and absorption at 60% RH is in the range of 2.4–2.5 percentage points for each of the three tissues, with moisture content being higher during desorption than during absorption.

### Axially dependent hygroscopic tissue shrinkage

Geometric differences between the tissue samples were observed using DVC to analyze the axial shrinkage from moist to dry state (Table 1). In the following, radial shrinkage (ε*_xx_* and ε*_yy_*) corresponds to shrinkage along both the abaxial–adaxial and the lateral–medial axis of a pine cone scale, since differentiation is not possible based on our tissue samples No statistical testing was performed to compare axial strains of individual tissue samples. The large number of observed voxels (>10^7^) could produce misleading significant results, even for negligible biological differences (see the Supplementary information in [29] for an example).

**Table 1:** Measured median axial strain across all evaluated voxels of the sample bulk.

tissue sample	sampling position	median axial strain with IQR			number of evaluated voxels

		ε*_xx_* [%]	ε*_yy_* [%]	ε*_zz_* [%]	

sclerenchyma fiber	adaxial	−8.6 (−14.0, −2.4)	−9.0 (−14.1, −2.9)	−1.8 (−7.8, 3.3)	125,594,142
sclerenchyma fiber	abaxial	−11.0 (−17.7, −2.9)	−8.6 (−16.4, 0.5)	−3.1 (−10.1, 3.9)	38,983,712
brown tissue	—	−10.5 (−14.4, −6.1)	−5.8 (−9.8, −1.4)	−7.6 (−10.8, −3.1)	56,858,642
sclereid cells	adaxial	−7.5 (−12.7, −2.5)	−7.6 (−12.9, −2.4)	−8.8 (−12.6, −5.4)	125,224,286
sclereid cells	abaxial	−6.7 (−14.3, 2.2)	−8.7 (−15.3, −1.5)	−8.1 (−13.0, −2.9)	145,464,169

The radial shrinkage of the cell samples ranged from the lowest of −5.8% for the brown tissue *y*-axial shrinkage to the highest of −11.0% for the sclerenchyma fiber *x*-axial shrinkage. The *x*- and *y*-axial shrinkage of the samples differed most for the brown tissue sample with the median *y*-axial shrinkage being 45% lower than the *x*-axial shrinkage (4.7 percentage points). On average, the radial shrinkage (average across sampling position and *x*- and *y*-axis for each tissue) of the sclerenchyma fibers was −9.3%, of the brown tissue −8.2%, and of the sclereid cells −7.6%. For longitudinal shrinkage (ε*_zz_*), the lowest value was measured for the abaxial and adaxial sclerenchyma fibers, with higher shrinkage in the abaxial fiber (abaxial: −3.1%; adaxial: −1.8%). The highest longitudinal shrinkage was measured for the sclereid cells, with the adaxial shrinking more than the adaxial (abaxial: −8.1%; adaxial: −8.8%). The brown tissue sample showed higher longitudinal shrinkage (−7.6%) closer to the sclereid cell layer than to the sclerenchyma fibers. The radial shrinkage of the adaxial sclerenchyma fibers is, for example, 4.8–5.0 times higher than the longitudinal shrinkage. Whereas for the radial shrinkage of the abaxial sclereid cells is 0.8–1.1 times the longitudinal shrinkage.

### Hygroscopic bending motion

The ten scales took 9.1 ± 1.6 h to reach their bending angle plateau from laboratory conditions to low RH (Figure 4B). From low RH to high RH, they took 10.0 ± 1.4 h to reach a plateau with a maximum measured angle of 17.8°. Regarding a correlation between their respective position along the central cone axis and their time until equilibrium, we cannot assume a normal distribution (Shapiro–Wilk test for high RH results: *W* = 0.936, *p* > 0.05, *n* = 10). Therefore, we calculated the Spearman’s rank correlation coefficient and found no significant correlation neither at low RH (rho = −0.161, *p* > 0.05) nor high RH (rho = 0.283, *p* > 0.05, *n* = 10). Comparing the linear model with an intercept-only (constant) model using AICc indicated a marginal preference for the constant model (ΔAICc = AICc_linear_ − AICc_constant_; low RH: ΔAICc = 1.38; high RH: ΔAICc = 0.09). As both ΔAICc values are ≤2 (i.e., models have comparable support), we chose the more parsimonious representation and report the mean and standard deviation (Figure 4C).

Measuring the opening angle as a function of relative humidity (Figure 4D) shows a dependence of the bending on the direction of the humidity change, and a difference in the angle obtained with respect to the position along the central axis. In particular, the opening angles are smaller for absorption than for desorption for the same observed climatic conditions. The difference between the angles is greatest in the range between 50% and 60% RH, reaching, for example, 11.9° for one of the most basal scales. When the angle is considered as a function of the position along the central axis, basal scales reach an angle of ~50°, while apical scales sometimes reach only ~20° (Figure 4E). For both ab- and desorption and all RH steps, we calculated the Spearman’s rank correlation between the normalized angle and the position along the central axes. We found significant correlation for all combinations (rho < −0.6, *p* < 0.05).

### Scale bending simulation

**Figure 5:**
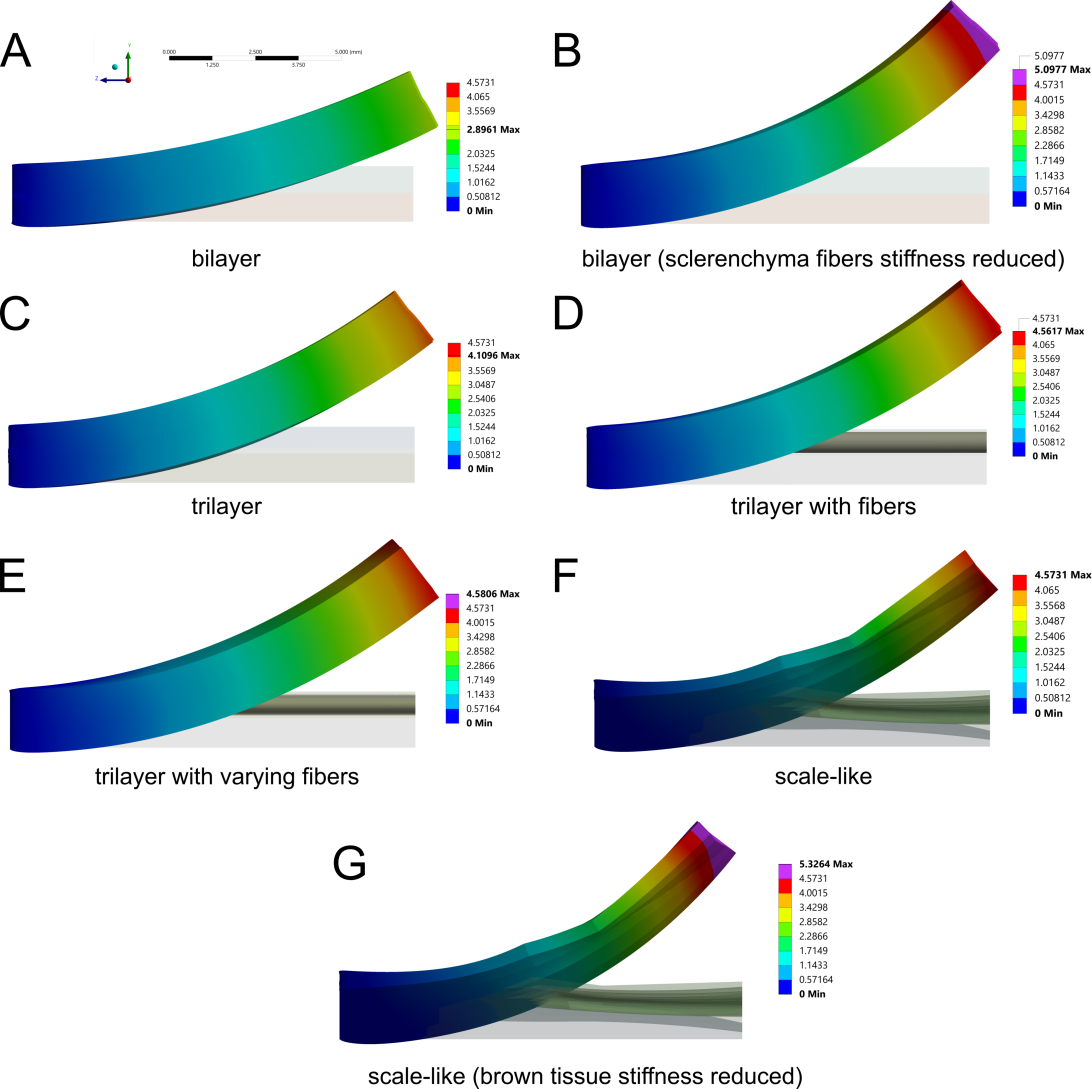
Results of the FE analysis. Obtained bending of (A) the bilayer, (B) the bilayer with reduced stiffness of the sclerenchyma fiber layer, (C) the trilayer, (D) the trilayer with fibers, (E) the trilayer with fibers with varying diameter, (F) the remodeled scale-like geometry, and (G) the remodeled scale-like geometry with reduced stiffness of the brown tissue. All results share the same coordinate system and scale depicted in (A). The colors indicate the total displacement.

The simulations of all five models resulted in a bending along the *Z*-direction of the geometry (Figure 5, Supporting Information Files 3–9). The largest bendings of the standard simulations were achieved by the trilayer with uniform fibers (maximum total displacement: 4.56 mm), the trilayer with varying fiber diameters (4.58 mm) and the scale-like geometry (5.57 mm). In the fiberless trilayer, a medium bending magnitude was achieved (4.11 mm), and the standard bilayer geometry bent the least (2.90 mm). By reducing the Young’s modulus of the sclerenchyma fiber layer in the bilayer, the maximum total displacement was increased to 5.10 mm. A similar trend was found for the scale-like geometry in which the Young’s modulus of the brown tissue was reduced, with an increased maximum displacement of 5.33 mm, and thus the highest value measured in any of the simulations. Looking at the change in curvature of the lateral axis of the geometries, a bending was particularly evident in the trilayer geometry with graded fibers and both scale-like geometries, while it was only slightly prominent in the other geometries (Figure S2b, Supporting Information File 2). In the case of the trilayer geometry with graded fiber diameters, the adaxial surface visibly bulged upwards. The pre-curved adaxial surface of the scale-like geometry flattens. This lateral curvature is influenced by the chosen radial expansion coefficient of the simulations, as highlighted in the comparative simulations of the geometry with graded fiber size (Figure S3, Supporting Information File 2). A lower expansion coefficient resulted in negligible lateral curvature, while a higher expansion coefficient resulted in increased lateral curvature.

To validate the FE simulations, a mesh sensitivity analysis was performed based on the trilayer geometry with fibers. Further mesh refinement with an increased number of nodes by a factor of ~3 resulted in a relative change in maximum deformation of only around 0.1% (For the mesh sensitivity analysis, elements sizes were changed from 0.15 mm (fibers) and 0.1 mm (sclereid layer and brown tissue) to 0.1 and 0.067 mm, respectively. The resulting mesh comprised 3,927,014 nodes and 2,681,955 elements. The maximum deformation of the scale changed from 4.5617 to 4.5574 mm, corresponding to a relative change of about 0.1%). Thus, mesh convergence can be assumed. Additional analysis of the impact of expansion coefficient variance based on the “trilayer with fibers” model showed the highest impact on bending for the *z*-axial hygroscopic expansion coefficient of the sclereid layer. More detailed results can be found in Table S1, Supporting Information File 2.

## Discussion

Our analysis of axis-dependent swelling and shrinkage revealed inter- and intra-tissue gradients of the hygroscopic strain. We observed the lowest longitudinal shrinkage (median: −1.8%) for the adaxial sclerenchyma fiber tissue. Additionally, we found a difference between the adaxial and abaxial sides concerning the extent of longitudinal shrinkage, with the abaxial side exhibiting about 70% greater median shrinkage (Table 1). This gradient is consistent with spiral secondary cell wall thickenings only being observed on the abaxial side, leading to a differential longitudinal extension rate within the sclerenchyma fibers measured using environmental scanning electron microscopy (abaxial: 9%, adaxial: 1%, [18]). Although their exact values of measured longitudinal expansion may differ from ours due to differences in methodology and pine species studied, our results do support the ability of individual fibers to bend independently in response to moisture changes due to an intra-tissue gradient. In contrast, the longitudinal shrinkage measured in the abaxial and adaxial sides of the sclereid layer was more uniform, with about 0.7 percentage points higher values for the adaxial side, likely being within the measurement noise. The brown tissue exhibited lower hygroscopic shrinkage than the sclereid layer, although not as low as that observed in the sclerenchyma fibers. The boundary between the sclereid layer and the brown tissue is gradual rather than discrete [24], suggesting a continuous gradient in hygroscopic behavior. This gradual transition also hinders identification of specific tissues during sample preparation, leading to the sample labeled as “brown tissue” possibly partially reflecting the characteristics of the adaxial sclereid layer. Additionally, our analysis was limited by the measurement of only a single sample per internal tissue sampling location. In order to refine our understanding of the hygroscopic material properties of tissue samples, future studies should include larger sample sizes. Nevertheless, the use of DVC-assisted sample evaluation has proven to be a very useful complementary method to classical ways for analyzing and comparing the hygroscopic shrinking behavior of lignocellulosic tissue samples.

When examining the radial tissue shrinkage (along the abaxial–adaxial and lateral–medial axes), we observed the intrinsic anisotropy of cell wall shrinkage [25,29,45]. Considering the *x*-axial shrinkage within the *x*–*y*-plane, cell walls oriented parallel to this axis have a lower *x*-axial shrinkage than perpendicularly oriented cell walls. The same can be observed for the *y*-axial shrinkage. This observation explains the variance between *x*- and *y*-axial shrinkage. Although under ideal sample preparation, the proportion of parallel and perpendicular cell wall sections should be similar (see adaxial sclerenchyma fibers), this is not always the case under real conditions. The porous brown tissue is a good example for this issue, showing high variance between the median *x*- (−10.5%) and *y*-axial (−5.8%) shrinkage. Therefore, future measurements should include more cells to avoid conflicting results for the radial shrinkage. In addition, the original orientation during specimen preparation and imaging should remain traceable in order to differentiate between ab-/adaxial and lateral directions in the resulting images. The tissue average radial shrinkage is highest for the sclerenchyma fibers and lowest for the sclereids, which can be explained by the microfibril angle. The angle of the microfibrils can not only limit cell expansion along its longitudinal direction, but also constrict radial shrinkage like a belt [46–47]. Since the microfibril angle is highest in sclereid cells, their longitudinal expansion is higher and their radial expansion is lower. The sclerenchyma fibers, in contrast, have a lower microfibril angle and expand less longitudinally and more radially.

Like the inter-tissue gradient of the hygroscopic strain, we also observed a clear hierarchy between the three tissues in terms of moisture content as a function of relative humidity (Figure 4). At the initial 90% RH before drying, the sclereid layer exhibited the highest moisture content (27.7%), followed by the brown tissue at a slightly lower level (25.5%), and the sclerenchyma fibers with the lowest moisture content (23.7%). This resulted in a moisture uptake profile that diverges notably from that reported by Eger and colleagues [23]. Comparing the water uptake measured by us in the range of 30–80% RH with that reported by Eger et al. [23], the behavior of the sclerenchyma fibers is comparable. However, both the sclereid layer and the brown tissue in our study exhibit a 1.5–2 times higher relative water uptake, a discrepancy that may arise from differences in the equalization criterion or inherent species-specific characteristics. It is important to note that different species were studied in each case, which may indicate that the water uptake of the tissues differs markedly between pine cones of different species.

Furthermore, our measurements reveal a pronounced hysteresis between absorption and desorption cycles in all three tissue types, indicating that the moisture content, and consequently the swelling behavior, is strongly dependent on the sample’s exposure history. A comparable hysteresis was also indicated in previous studies [23–24], but has not yet been investigated further. This path-dependency may affect the repeatability of hygroscopic behavior under fluctuating environmental conditions. The same phenomenon can be observed when measuring the bending angle of the cone scales during absorption and desorption (Figure 4). Within the 50–60% RH range, the bending angle varied markedly depending on the pre-conditioning of the scale: Scales that were drier prior to exposure achieved up to ~12° smaller bending angles than those that were previously moistened. This observed hysteresis of the scale bending angle is closely related to the sorption hysteresis of the scale tissues and may also have biological implications. Considering a cone has just opened and is releasing its seeds, it would be disadvantageous for the cone scales to “overreact” and close due to every slight rain shower or increase in humidity. In this context, the hysteresis might act as an additional buffer besides the general slow speed of the closure. Delayed closure relative to humidity after a dry state ensures that the cone does not respond abruptly to transient increases in humidity, thereby stabilizing the seed dispersal process. A similar mechanism may apply to closed cones, where it has already been described that the initial opening is influenced by resin at the apophysis and is therefore dependent on temperature and humidity being within a certain range [20]. After the initial opening, a delayed response to humidity changes could additionally prevent inadvertent responses to transient humidity decreases, ensuring that the cone only opens when environmental conditions consistently favor seed dispersal. This should also be taken into account and further investigated in the case of future studies, not only to enhance the understanding of the ecological importance of the pine cone opening and closure, but also to improve the design of bioinspired systems. Controlled, delayed responses to environmental changes may be desirable for various engineering and architectural systems, such as adaptive façade shading systems [15].

The achievable bending angle also varied with the position of the scales along the central cone axis. Scales from the apical part achieve an up to ~30° lower opening angle than those from the basal part. This may be attributed to variations in tissue composition or mechanical and hygroscopic properties along the cone. In the case of basal scales, our measured opening angle is underestimated due to the chosen method of angle calculation and the strong curvature of the scales. Interestingly, we found that the time required for the scales’ bending motion to reach an equilibrium was consistent across different positions along the cone. This uniformity suggests that the time until equilibrium may be independent of local structural variations, potentially ensuring coordinated movement across the cone. In this case, if evaporation through the apophysis were the primary driver of moisture loss in a closed cone, all scales would be expected to open simultaneously. In *Pinus jeffreyi*, however, the orchestration occurs successively. This supports the conclusions drawn by Horstmann et al. [21], who attributed the successive orchestration to the overlapping arrangement of scales and the constrained evaporation through the apophysis. This coordinated opening mechanism may facilitate seed dispersal since not all seeds are released at the same time. It also provides valuable insights for the design of bioinspired systems that mimic such controlled, sequential responses and actuations.

The results of the simulations clearly show that we can use the measured values of hygroscopic expansion to achieve a bending angle in the range of biological specimens (Figure 5). However, the chosen material properties of the tissues, and in particular the cross-sectional area of the more rigid sclerenchyma fiber layer, play a crucial role. For example, the simulation of the bilayer geometry showed that a considerably lower bending is achieved compared to other geometries. This is due to the cross-sectional geometry and the shape and proportion of the stiffer sclerenchyma fiber layer. When the cross-sectional geometry is simplified to a bilayer with the sclerenchyma fibers as a rectangular layer, the resulting area moment of inertia increases compared to a layer consisting of multiple fibers. Thus, the sclerenchyma fiber layer contributes more to the structural Young’s modulus of the cone scale model, and the resulting bending is less than that observed in nature. Since previous simulations of the pine cone scale have used the bilayer model as a simplified geometry [23,30], the obtained results must be taken with caution. However, if the sclerenchyma fibers’ Young’s modulus is decreased, or if they are considered as discrete fiber elements embedded in an intermediate tissue, higher bending angles can be achieved with the same material properties and more closely represent the biological model.

As part of these considerations of the relevance of Young’s modulus, it was also noted that the indentation measurements by Eger et al. [23] used as the basis for our simulations may reflect a cellular rather than a structural Young’s modulus of the tissue. In their measurements, the Young’s modulus of the brown tissue is in the same range as that of the sclereid layer. This is despite the fact that in cone scales of *P. jeffreyi* and other species, for example, the brown tissue is significantly more porous [24] than the sclereid layer and can be easily deformed and bent by hand, while the sclereid layer remains relatively rigid. This porosity can also be seen in the cross sections we prepared (Figure 1) and in the CT images (Figure S4, Supporting Information File 2). Since the choice of probe tip for nanoindentation measurements of hierarchically structured biological samples is very important in relation to what one wants to measure [48–49], it is possible that a tip that is too small was used. It is also possible that the gradual transition between sclereid cells and brown tissue may have caused the indentation to sample predominantly the adaxial sclereid cells rather than the brown tissue.

Our simulations not only extend the geometric complexity of previous bilayer models [23,30]; they also take into account the measured axial swelling instead of assuming isotropy. This leads not only to curvature along the longitudinal axis, but also to lateral curvature, albeit less pronounced. In contrast to the biologically observed opening motion [12], the lateral curvature we observe is inverted. This effect can be explained by the higher average radial shrinkage/swelling of brown tissue compared to sclereid cells. This may be due to the higher measurement variance of the DVC analysis due to the porous structure of the brown tissue. When the simulations are performed with the lower *y*-axis shrinkage, this lateral curvature is negligible, whereas when the higher *x*-axis shrinkage is chosen, the inverted movement becomes even more pronounced (Figure S3, Supporting Information File 2). This illustrates the influence of the radial shrinkage/swelling properties of the motions involved and the measurement errors on the radial curvature. Furthermore, only a single parameter was varied in the comparative simulation, so it can be assumed that the biological curvature can be reproduced by further variations. However, the aim of this work was not to adjust the individual parameters of the simulation to achieve the best possible agreement between experiment and simulation, but rather to set up a simulation based on the experimentally determined values and to use it to characterize the influence of the underlying geometries and tissue distributions. Our analysis of the impact of parameter variation on achieved bending highlighted the importance of *z*-axial hygroscopic expansion of the sclereid layer. This finding further supports the results of our simulations based on DVC-estimated hygroscopic expansion coefficients. While the simulations are sensitive to changes in the *z*-axial hygroscopic expansion coefficient, their results are comparable to those of biological samples. However, further refinements to the DVC-based estimation are essential to strengthen the predictive reliability of upcoming simulations. For the analysis of the motion mechanism of the scale, for example, to show the multiphase nature of the motion [12], future simulation approaches could use transient heat transfer simulations incorporating diffusion models.

The comparative simulations in this work, together with previously published bilayer models, show that the analytical analysis of such a complex drying movement is a sensitive system of selected expansion coefficients, mechanical properties, and geometric models. It should be noted that the modeling of sorption/desorption of water via thermal expansion/contraction is common for plant structures due to the limitations of FEA software [23,50], but it involves an artificial detour. We have shown that the assignment of mechanical properties in multimaterial systems plays a crucial role in the extent of actuation; hence, the best possible data base is crucial for the accuracy and thus the informative value of the simulation. It should also be mentioned that most simulations assume clear boundaries between individual tissues/components and thus can only partially represent the natural models and their complex, often graded interfaces. Furthermore, our work has demonstrated the influence of the complexity of the simulated geometry. The geometries recently used to simulate plant systems range from highly simplified 2D geometries for complex damage analysis [51–52], to rather simplified rod-like structures [53] and 3D shell models [50,54], and ultimately to highly detailed geometries based on segmented computed tomography images [55–56]. No general guideline can be given for the level of detail required for FEA of plant systems as this depends on the complexity of the system, the available data, and, most importantly, the research question to be answered. The data presented should demonstrate the importance of considering geometries. It should be noted that simplifying each tissue as a homogeneous layer still does not fully represent biological samples. Our approach does not consider internal gradients and gradual interfaces, which can be observed in the samples. However, these features should be included when creating highly detailed virtual twins of the pine scales. Considering all of the above factors and the associated uncertainties, FEA analysis can have an ever-increasing impact on the biomechanical analysis and characterization of plant motion. To better describe and analyze the pine cone scale in silico in the future, the material properties of the tissues should be re-measured, focusing on their structural rather than cellular properties. A detailed mesh of a pine cone scale based on a segmented CT scan could also be the next step to overcome the limitations of the currently used simplifications.

## Conclusion

In summary, returning to our initial research questions, we were able to observe and further investigate inter- and intra-tissue gradients of hygroscopic swelling/shrinkage and moisture content. In addition, we observed hysteresis both at the tissue level with respect to moisture content and in the measurements of the bending angle of the scales as a function of relative humidity. The measured hygroscopic expansion coefficients were used for FEA of various geometries resembling simplified pine cone scale bending zones. This analysis highlighted the importance of the tissue material properties and raises some questions about previous Young’s modulus measurements. Furthermore, the achievable bending angle can be increased by avoiding a simple bilayer geometry and modeling the sclerenchyma layer as discrete fibers embedded in a brown tissue matrix. Therefore, the use of more complex geometries, such as a remodeled scale, is recommended for a more accurate study of the biological system in silico.

## Supporting Information

File 1 Detailed parameter files for digital volume correlation. The ZIP archive contains two txt files, that is, “Affine_parameters.txt”, the parameter file for affine registration, and “BSpline_parameters.txt”, the parameter file for B-spline registration.

File 2 Additional experimental data.

File 3 Bending simulation result of the bilayer model.

File 4 Bending simulation result of the trilayer model.

File 5 Bending simulation result of the fiber model.

File 6 Bending simulation result of the fiber model with a gradient.

File 7 Bending simulation result of the scale-like model.

File 8 Bending simulation result of the bilayer model with reduced E-modulus.

File 9 Bending simulation result of the scale-like model with reduced E-modulus.

## Data Availability

Data generated and analyzed during this study is openly available in Mendeley Data at https://doi.org/10.17632/phyx35hcy6.1. Due to imaging data size, additional research data generated and analyzed is available from the corresponding author upon reasonable request.
