# Supplementary material for: Beyond the bilayer: multilayered hygroscopic actuation in pine cone scales
Source: Beilstein J Nanotechnol. 2025 Sep 29;16:1695–710. doi: 10.3762/bjnano.16.119 (PMC12498252; doi:10.3762/bjnano.16.119)
Supplement: File 2 — Additional experimental data. [file Beilstein_J_Nanotechnol-16-1695-s002.pdf]

BEILSTEIN JOURNAL OF NANOTECHNOLOGY

Supporting Information

for

Beyond the bilayer: multilayered hygroscopic actuation in pine cone scales

Kim Ulrich, Max David Mylo, Tom Masselter, Fabian Scheckenbach, Sophia Fischerbauer, Martin Nopens, Silja Flenner, Imke Greving, Linnea Hesse and Thomas Speck

Beilstein J. Nanotechnol. **2025,** *16,* 1695–1710. doi:10.3762/bjnano.16.119

Additional experimental data

License and Terms: This is a supporting information file under the terms of the Creative Commons Attribution License (https://creativecommons.org/licenses/by/4.0). Please note that the reuse, redistribution and reproduction in particular requires that the author(s) and source are credited and that individual graphics may be subject to special legal provisions.
The license is subject to the *Beilstein Journal of Nanotechnology* terms and conditions: (https://www.beilstein-journals.org/bjnano/terms)

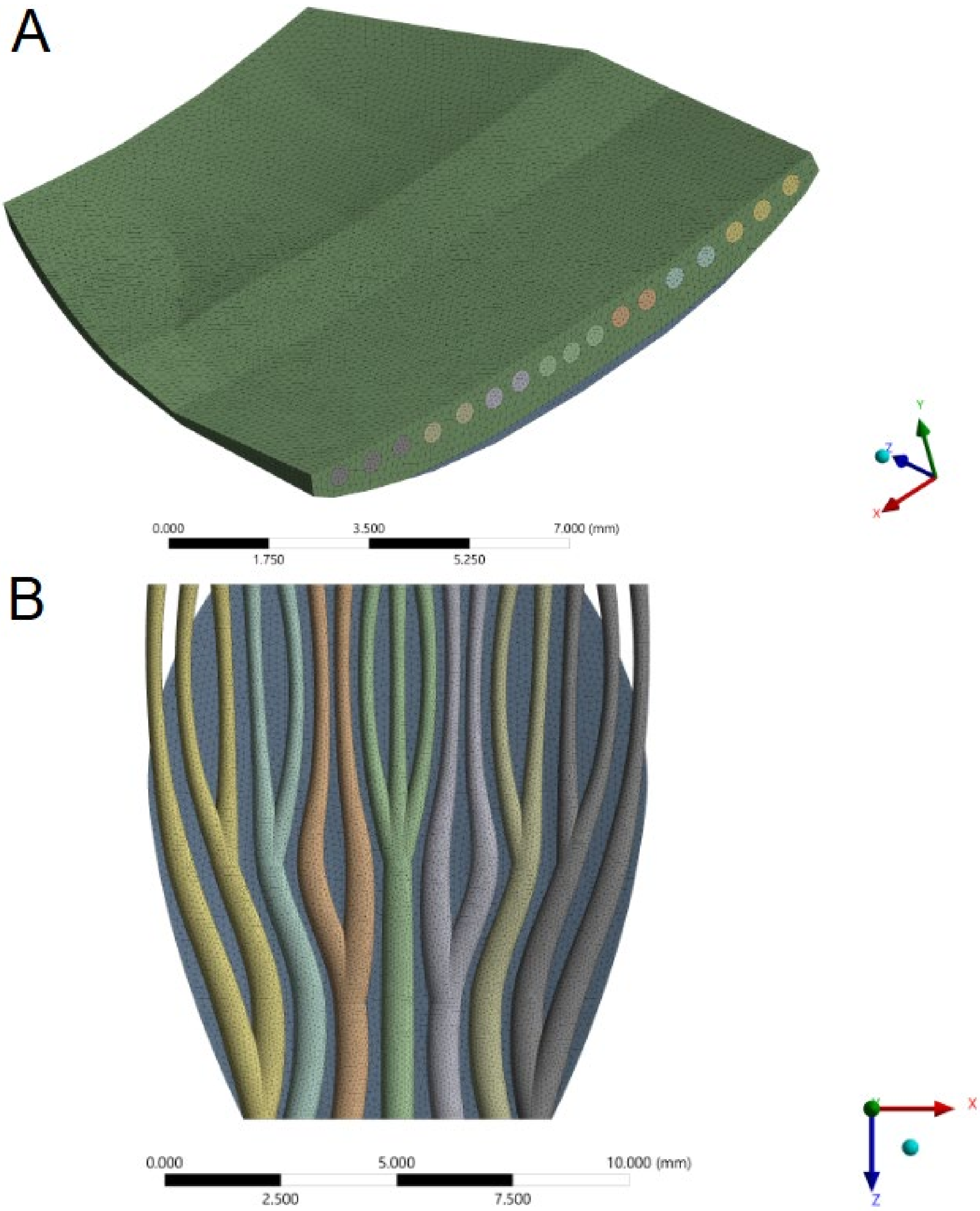

Figure S1: Resulting volume mesh of the scale-like geometry, including sclereid layer (gray layer), brown tissue (green layer), and sclerenchyma fibers (colored per branching strand). (A) Angled top-down view from apical direction. (B) Top-down view of the sclerenchyma fiber network with the brown tissue layer hidden.

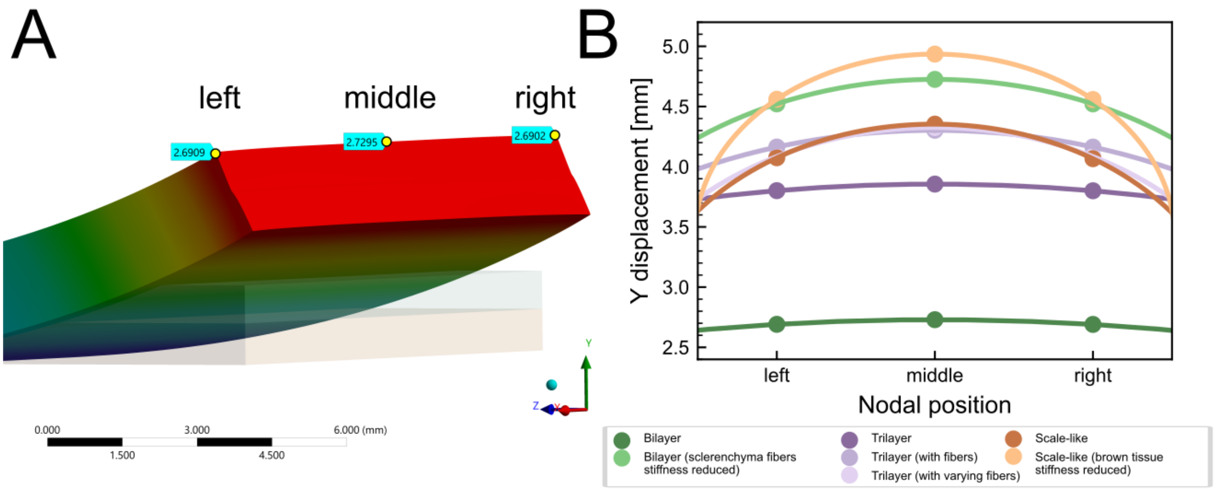

Figure S2: Estimation of the projected curvature based on the *y*-axial displacement. (A) The nodal positions (left, middle, right) at the apical front surface from which the displacement values were extracted from for each FEA are marked with yellow points in the result of the bilayer geometry. (B) Based on the *y*-axial displacement, the change in curvature along the lateral axis (*x*-axis) is estimated for each geometry.

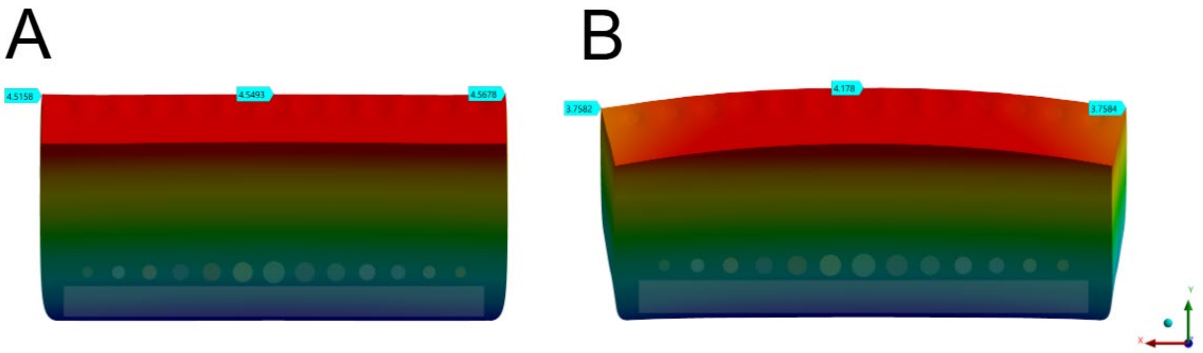

Figure S3: Comparative simulation of the geometry with graded fiber size. Front view of the simulations with (A) lower and (B) higher expansion coefficient of the brown tissue. The highlighted nodal positions (left, middle, and right) at the apical front surface show the displacement values.

Table S1: Simulations of the varied axial expansion coefficient with the resulting maximum displacement.

Axial expansion coefficient						
Sclereid fibers			Sclereid layer			Max. displacement [mm]
X	*Y*	*Z*	*X*	*Y*	*Z*	
0.001025	0.001025	0.000250	0.000825	0.000825	0.000920	4.561
0.001025	0.001025	0.000250	0.000900	0.000900	0.000920	4.633
0.001025	0.001025	0.000250	0.000660	0.000660	0.000920	4.378
0.001025	0.001025	0.000250	0.000825	0.000825	0.001100	5.848
0.001025	0.001025	0.000250	0.000825	0.000825	0.000740	3.282
0.001230	0.001230	0.000250	0.000825	0.000825	0.000920	4.409
0.000820	0.000820	0.000250	0.000825	0.000825	0.000920	4.698
0.001025	0.001025	0.000300	0.000825	0.000825	0.000920	4.303
0.001025	0.001025	0.000200	0.000825	0.000825	0.000920	4.819

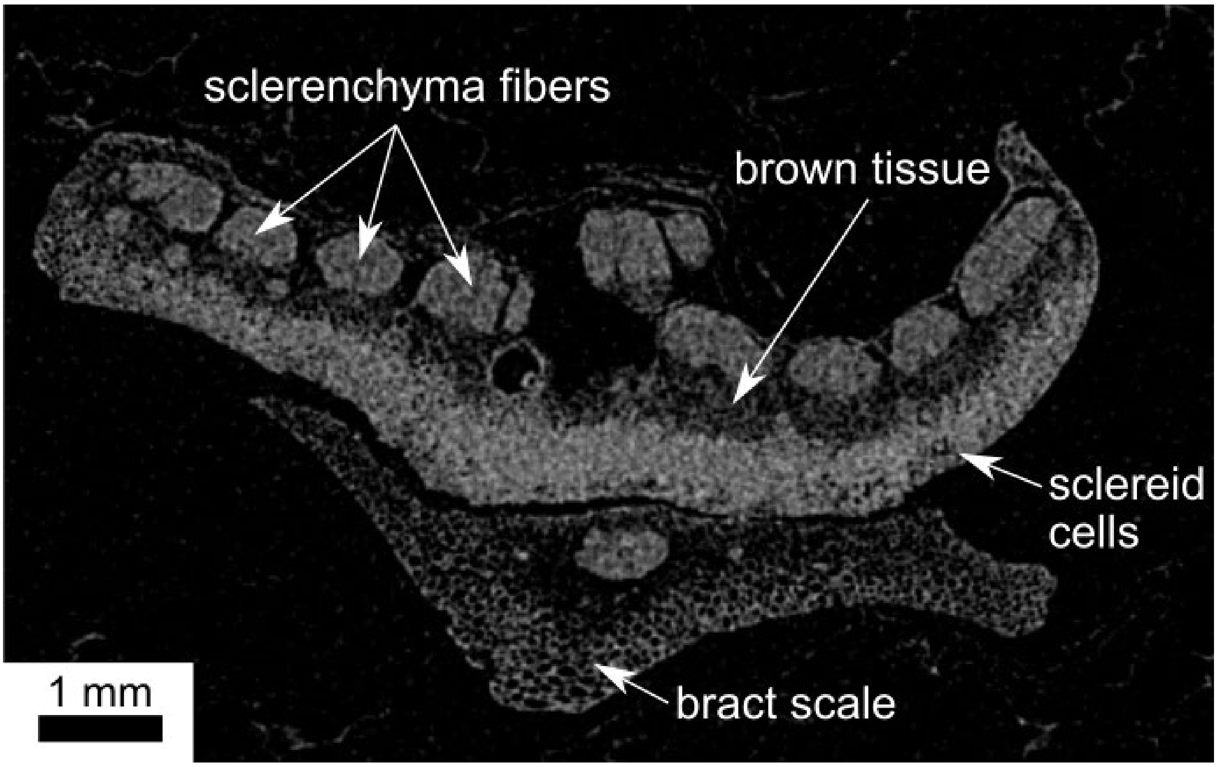

Figure S4: Microtomographic cross-sectional image of a pine cone scale of *Pinus jeffreyi*.
